# Comparison of the effectiveness of virtual reality and chewing mint gum on labor pain and anxiety: a randomized controlled trial

**DOI:** 10.1186/s12884-021-04359-3

**Published:** 2022-01-19

**Authors:** Atefeh Ebrahimian, Roghaieh Rahmani Bilandi, Mohammad Reza Rahmani Bilandī, Zahra Sabzeh

**Affiliations:** 1 Department of Midwifery, Faculty of Medicine, Social Development & Health Promotion Research Center, Gonabad University of Medical Sciences, Gonabad, Khorasan Razavi Iran; 2 School of Nursing & Midwifery, Ahvaz Jundishapur University of Medical Science, Ahvaz, Iran; 3 Department of Midwifery, Faculty of Medicine, Gonabad University of Medical Sciences, Gonabad, Khorasan Razavi Iran; 4 Department of Midwifery, Gonabad University of Medical Sciences, Gonabad, Khorasan Razavi Iran

**Keywords:** Labor pain, Anxiety, Virtual reality, Chewing gum

## Abstract

**Objective:**

Childbirth pain and anxiety are often unnatural, as opposed to being one of the most practical ways to use non-pharmacological methods. The aim of this study was to compare the effectiveness of virtual reality and chewing mint gum on childbirth pain and anxiety.

**Methods:**

This is a single-blind, three-group clinical trial study on 93 mothers referred to Allameh Bohlool Gonabadi and Sajjadieh Torbate Jam Hospitals for natural childbirth in 2018–2019. Subjects were randomly divided into three groups of chewing gum, virtual reality, and control using six blocks. Chewing gum interventions in one group and virtual reality in the other group were performed twice in 4–5 cm and 7–8 cm dilatations for 20 min. In the control group, no intervention except routine care was performed. The research tools included Visual Analogue Scale of Pain and Spielberger’s Anxiety Inventory. Data were analyzed using SPSS) version 22(, ANOVA, Kruskal–Wallis, Chi-square and Tukey tests. Significance level was considered 0.05 in this study.

**Results:**

The main result was differences in pain and anxiety before and after the intervention. There was no significant difference between pre-intervention pain and anxiety scores in the three groups, but there was a significant difference between pain and anxiety scores immediately and 30 min after the intervention.

**Conclusion:**

The results of this study showed that virtual reality and chewing mint gum intervention reduce pain and anxiety in the first stage of childbirth.

**Trial registration:**

IRCT20181214041963N1.

## Introduction

Childbirth has been described as a painful physiological phenomenon throughout women’s lives. Pain is an unpleasant condition that the endeavor of medical science has always sought to eliminate or reduce [1]. Fear of childbirth is a major women’s resistance to natural childbirth and therefore women prefer cesarean childbirth [2, 3]. Studies show that about 60% of nulliparous women and 40% of multiparous women describe severe childbirth pain [4]. Anxiety due to anxiety with increased epinephrine and norepinephrine causes vasoconstriction, increased muscle tone, decreased uterine contractility, and abnormalities of childbirth [5]. Factors such as stress, uneducated, and unawareness and lower socioeconomic status can cause severe pain. Adverse effects of severe pain on the neonate can be attributed to the late decline in heart rate due to decreased maternal arterial oxygen pressure, low Apgar score, fetal heart sound disorder, uterine-placental abnormality due to severe uterine contractility and sometimes fetal acidosis [6]. Anxiety and pain are two important factors that contribute to the process of childbirth [7]. The effect of anxiety on childbirth progress, increased bleeding during childbirth, exacerbation of childbirth pain, negative experiences of childbirth, mental problems and postpartum depression and delayed onset of first breastfeeding [8]. The severity of pain and anxiety during childbirth depends on mother’s mental stress and identification and coping with them are the most practical ways of using non-pharmacological methods [9, 10]. These include aromatherapy, distraction, acupuncture, acupressure, reflexology, respiratory techniques, massage therapy, and music therapy, etc. [11]. One of the non-pharmacological approaches to reducing pain and anxiety is the use of the distraction technique, which nowadays utilizes a variety of distraction techniques in painful medical processes. The distraction is more in line with the painful tendency, and the negative process, such as pain, is on the margin of attention reduces the feeling and perception of pain. Two ways of viewing virtual reality films and chewing gum are subsets of distraction. Virtual reality reduces attention to real stimuli by placing special glasses containing 360-degree relaxation films in front of one’s eyes [12]. It has recently been used as a method to control pain and anxiety during medical procedures [13]. One study stated that virtual reality can provide effective distraction for people with pain from a variety of physical and mental illnesses [14]. Chewing gum can also reduce stress and environmental anxiety by creating distraction. A study of the role of chewing gum on stress, consciousness, and cognition suggests that chewing gum is effective in reducing chronic stress and acute stress [15]. Studies show that chewing mint gum is more effective in reducing anxiety than chewing other chewing gum [16], so in this study, the taste of mint gum was used. According to the results, the preferred methods for reducing pain and anxiety are methods that are available, easy to use, do not require expertise and are under the control of the individual, so the need for this study is determined. Studies have shown that both methods of distraction (virtual reality and chewing gum) are somewhat effective in reducing pain and anxiety in medical practice. But it is not possible to say which of the two methods of distraction is preferred. The aim of this study was to compare the effect of virtual reality and chewing mint gum on labor pain and anxiety.

## Method

### Trial design

This study was designed as a clinical trial and was registered at the Iranian Registry of Clinical Trials on 27/12/2018 (IRCT20181214041963N1). The report of this clinical trial is based on the CONSORT 2010 checklist [17].

### Participants and setting

This study is a Main part of the findings of a larger study examining the effects of labor pain and anxiety. This randomized clinical trial with three parallel groups was performed on 93 mothers in Allameh Bohlool Gonabad and Sajadieh Torbate Jam Hospital in Iran (protocol of labor in two center was similar). Sampling was done from February 2018 to June 2019. Inclusion criteria included: consent to participate in the study, gravida 1or 2, singleton pregnancy with live embryo, entry into active phase of childbirth, maternal age between 18 and 35 years, pregnancy stage 37–41 weeks, lack of medical and mental illness, lack of abnormal embryos, cephalic manifestations, low risk pregnancy, healthy caul or less than 8 h have passed since their rupture, no history of motion sickness, no blindness, no addiction, and estimated weight of the fetus up to 4000 g. Exclusion criteria were: mother’s unwillingness to cooperate, midwifery problems, use of Entonox and spinal and epidural anesthesia, chewing gum less than 20 min and watching virtual reality film less than 20 min.

### Sample size and randomization

Sample size was calculated using G-Power software based on numerical methods to obtain n number of samples in k group (k = 3). An effect size of f was equal to 0.35% of the first type error of maximum 5%, test power 80% and number of comparison groups was set at three, which required a sample size of 84. The final sample size was 93 mothers in three groups with 10% fall.

$$\frac{\sum \left(\overline{{\boldsymbol{y}}_{\boldsymbol{L}}}-\overline{{\boldsymbol{y}}_{..}}\right)/\left(\boldsymbol{k}-\mathbf{1}\right)}{\sum \sum \left({\boldsymbol{y}}_{\boldsymbol{ij}}-\overline{{\boldsymbol{y}}_{\boldsymbol{L}}}\right)/\left(\boldsymbol{n}-\boldsymbol{k}\right)}-{\boldsymbol{F}}_{\mathbf{1}-\boldsymbol{\alpha}}\left(\boldsymbol{k}-\mathbf{1},\boldsymbol{n}-\boldsymbol{k}\right)>{\boldsymbol{F}}_{\mathbf{1}-\boldsymbol{\beta}}\left(\boldsymbol{k}-\mathbf{1},\boldsymbol{n}-\boldsymbol{k}\right)$$

Sampling was done Convenience Sampling method and 6 blocks were used for random allocation of samples to each group. 6 possible modes are listed for the blocks and assigned to each block a number from 1 to 6 and randomly selected a number between 1 and 6, followed by individuals based on the block corresponding to the number selected to the virtual reality group (B), chewing gum group (C) and control (A) were assigned. Mothers and researchers were not blind to the intervention, while the statistical analyst who performed the data analysis was one-blind.

### Study instruments

Data collection tools included demographic and fertility characteristics questionnaires, visual analogue of pain and Spielberger’s anxiety questionnaire. The demographic questionnaire was provided to ten faculty members for review and corrective comments were applied after the survey. The visual analogue of pain is a 10-mm line that the beginning and end of it are denoted by the numbers 0 and 10. So that zero means pain and number 10 is the most severe pain that one feels in the lower abdomen and back. The Visual Anxiety Scale is a standard tool and its validity and reliability have been globally proven (r = 0.88) [18]. The Spielberger’s Anxiety Inventory is a standard questionnaire that consists of 20 items of explicit anxiety section. In the range of 4 Likert options, scores range from 1 to 4 (not at all, sometimes, generally, very much) and on a general scale, 20 to 80 are measured. A score of 20 indicates the lowest level of anxiety and a score of 80 indicates the highest level of anxiety. Each of the test terms is assigned a score of 1 to 4 based on the answer. A score of 4 indicates a high level of anxiety. Scoring is inverted for expressions that indicate anxiety. Depending on the item, the response scores will be assigned to 4–3–2-1 instead of 1–2–3-4. Phrases that indicate the absence of anxiety are reversed when scoring [19]. The validity of this questionnaire in Iran has been confirmed by Behrouz Mahram (1993). The reliability of the questionnaire was calculated by Cronbach anxiety ‘s alpha method of 0.889 [20].

### Interventions and outcomes

At the beginning of the study, after obtaining informed consent from the research units, a demographic and fertility questionnaire was completed by questioning Mother and her hospitalization file. Before the intervention, the researcher trained the use of pain visual analogue for three groups. Pain visual analogue and Spielberger Anxiety Questionnaire were completed by all research units before intervention. The chewing gum group was given a 1 g mint gum without sugar (with official permission from the Ministry of Health of Iran) in two stages, first at the beginning of the active phase (4 to 5 cm dilatation) and the second time at 7–8 cm dilatation to chew at their normal speed for at least 20 min. In the virtual reality group, virtual reality glasses containing 360-degree video with nature landscapes (Samsung Gear VR Virtual Reality Headset with Samsung Mobile S7) were performed twice, the first intervention was performed at the beginning of the active phase (4–5 cm dilatation) and the second intervention at 7–8 cm dilatation. Each intervention lasted for 20 min. The intervention groups (virtual reality and chewing gum) completed the pain visual analogue and the Spielberger Anxiety Inventory immediately after each intervention as well as the first 30 min after each intervention. In the control group, only routine maternity unit care was received according to the national protocol of midwifery care similar to the intervention groups. Routine delivery unit care Such as receive analgesia) oxytocin in the first and second stages of labor, misoprostol, pethidine, hyoscine, promethazine and atropine (except for the use of Entonox and spinal and epidural anesthesia, control fetal heart rate, vaginal exams, vital signs recording and more etc. Like the other two groups, pain visual analogue and anxiety questionnaire were measured simultaneously with the intervention group. All interventions in the two hospitals, including the implementation of virtual reality, chewing gum and recording the relevant questionnaires were performed by a postgraduate midwife who had received the necessary training in the morning, noon and night shifts.

### Statistical analysis

Data were analyzed by SPSS version 22 software. Data were analyzed using ANOVA and Kruskal-Wallis statistical tests and Chi-square test for qualitative data. Tukey was used to compare the two groups. Statistical analyst did not know the type of groups. Significance level was considered 0.05 in this study. Ethical considerations in this study include obtaining informed consent from research units by providing complete information about the intervention and control group, permitting exit from research at any stage, ensuring the confidentiality of the research data, fully observation of ethics, and trusting other research, and publishing accurate and real results.

## Results

**Fig. 1:**
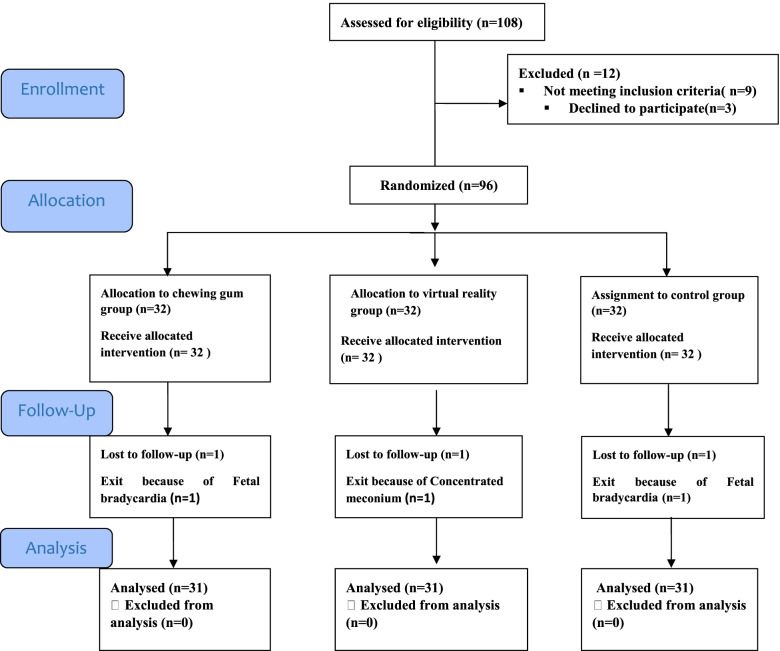
CONSORT diagram shows the flow of mothers

In this study, 108 individuals were evaluated for eligibility and 96 individuals were included in the study and allocated into three groups. Mothers in three groups from the active stage of labor to Postpartum stage was followed And 93 individuals (31 in the virtual reality group, 31 in the chewing Gum group and 31 in the control group) were evaluated (Fig. 1). The mean age of the mothers was (24/23 ± 4/44) years and the mean pregnancy stage was 39.31 weeks. The majority of mothers were nulliparous (65/6%) and mothers in the three groups had no significant difference in terms of oxytocin intake (*p* = 0.177) and other medications for accelerating and reducing pain. During the study, three patients were excluded from the active phase due to thick meconium and fetal bradycard. The study consort chart is shown in Fig. 1. The results showed that mothers in the three groups had no significant difference in demographic and midwifery information (Table 1). Kolmogorov-Smirnov test was used for data normality. The result of Kruskal-Wallis statistical test for prior pain, and 30 min after intervention in 4–5 cm dilatation in the study groups showed no significant difference between pain before and immediately after intervention in 4–5 cm dilatation in the three study groups but 30 min after intervention there was a significant difference in the three groups. According to Tukey’s test, the mean pain 30 min after the intervention was not significantly different between the two intervention groups (chewing gum and virtual reality) (*P* = 0.964), but the mean pain was significantly lower than the control group (*P* < 0.02).

**Table 1:** Comparison of individual and midwifery data in the three groups of study

Variable		virtual reality N (%)	Control N (%)	chewing gum N (%)
**Job**	**housewife**	**28 (90.32%)**	**31 (100%)**	**27 (87.09%)**
	**Student or employed**	**3 (9.68%)**	**0 (0%)**	**4 (12.90%)**
**Education level**	**Elementary school**	**0 (0%)**	**0 (0%)**	**2 (6.45%)**
	**Junior high school**	**11 (35.48%)**	**14 (45.16%)**	**12 (38.70%)**
	**Senior high school**	**13 (41.94%)**	**16 (51.61%)**	**9 (29.03%)**
	**University**	**7 (22.58%)**	**1 (3.23%)**	**8 (25.82%)**
**Socioeconomic Status**	**Poor**	**14 (45.16%)**	**19 (61.29%)**	**14 (45.16%)**
	**Moderate**	**17 (54.84%)**	**12 (38.71%)**	**17 (54.84%)**
**Pregnancy condition**	**wanted**	**28 (90.32%)**	**30 (96.77%)**	**27 (87.09%)**
	**Unwanted**	**3 (9.68%)**	**1 (3.23%)**	**4 (12.90%)**
**Participation in Maternal Readiness Class**	**Yes**	**9 (29.03%)**	**7 (22.58%)**	**13 (41.93%)**
	**No**	**22 (70.97%)**	**24 (77.42%)**	**15 (58.07%)**
**Gravida**	**1**	**23 (74.19%)**	**15 (48.38%)**	**16 (51.62%)**
	**2**	**8 (25.81%)**	**16 (51.62%)**	**15 (48.38%)**
**Parturition**	**0**	**25 (80.64%)**	**18 (58.07%)**	**18 (58.07%)**
	**1**	**6 (19.36%)**	**13 (41.93%)**	**13 (41.93%)**
**BMI**	**Underweight**	**0 (0%)**	**0 (0%)**	**0 (0%)**
	**Norma lweight**	**3 (9.68%)**	**8 (25.82%)**	**3 (9.68%)**
	**Overweight**	**24 (77.42%)**	**20 (64.50%)**	**27 (87.09%)**
	**Obesity**	**4 (12.90%)**	**3 (9.68%)**	**1 (3.23%)**

*analyzed by Chi-square test

In relation to pain before, immediately and 30 min after the intervention in 7–8 cm dilatation in the study groups, there was a statistically significant difference between pain before, immediately and 30 min after intervention in 7–8 cm dilatation. According to the Tukey test, there were no significant differences in pain before the intervention between the two groups (chewing gum and virtual reality) (*P* = 0.632), but the pain scores before the intervention were significantly lower than the control group (*P* < 0.008). Mean pain immediately after the intervention was not significantly different between the two groups (*P* = 0.862) but the pain scores were significantly lower than the control group (*P* = 0.000). Mean pain 30 min after the intervention was not significantly different between the two groups (*P* = 0.517), but the pain scores were significantly lower than the control group (*P* < 0.04) (Table 2).

**Table 2:** Comparison of mean scores of mothers’ pain severity in three groups of virtual reality, chewing gum and control *

Time	Variable	virtual reality	Control	chewing gum	***P*** value
		Mean ± SD	Mean ± SD	Mean ± SD	
	**Pain before intervention**	**6.25 ± 1.09**	**6.16 ± 1.34**	**6.19 ± 1.01**	***P*** **= 0.893**
**Dilatation 4-5 cm**	**Pain immediately after the intervention**	**6.70 ± 1.18**	**7.32 ± 1.24**	**6.74 ± 1.12**	***P*** **= 0.079**
	**Pain 30 min after intervention**	**7.22 ± 1.11**	**8.16 ± 1.26**	**7.32 ± 1.22**	***P*** **= 0.008**
	**Pain before intervention**	**8.37 ± 0.88**	**9.22 ± 0.71**	**8.58 ± 0.88**	***P*** **= 0.001**
**Dilatation 7-8 cm**	**Pain immediately after the intervention**	**9.03 ± 0.83**	**9.77 ± 0.49**	**8.93 ± 0.81**	***P*** **= 0.000**
	**Pain 30 min after intervention**	**9.61 ± 0.61**	**9.96 ± 0.17**	**9.45 ± 0.76**	***P*** **= 0.001**

*analyzed by Kruskal-Wallis statistical test

The results of ANOVA and Kruskal-Wallis statistical test for anxiety before, immediately after intervention and 30 min after intervention in 4–5 cm dilatation in the study groups showed that there was no significant difference between anxieties before intervention in 4–5 cm dilatation, but there was a significant difference between anxieties immediately after intervention in the three study groups, according to Tukey’s test, the mean anxiety immediately after the intervention was not significantly different between the two groups (chewing gum and virtual reality) (*P* = 0.849), but the mean anxiety was significantly lower than the control group (*P* < 0.001). There was also a statistically significant difference between 30 min after the intervention in the three study groups. According to Tukey’s test, the mean anxiety 30 min after the intervention was not significantly different between the two intervention groups (chewing gum and virtual reality) (P 0.847), but the mean anxiety was significantly lower than the control group (*P* < 0.001).

Results of ANOVA and Kruskal-Wallis test for anxiety before intervention, immediately after intervention and 30 min after intervention in 7–8 cm dilation in study groups showed that there was a statistically significant difference between anxiety before and after intervention in 7-8 cm dilatation in the three study groups, according to the Tukey’s test, the mean anxiety before intervention (7–8 cm dilatation) was not significantly different between the two intervention groups (chewing gum and virtual reality) (*P* = 0.488), but the mean anxiety was significantly lower than the control group(*P* < 0.001). There was also a statistically significant difference between anxieties immediately after the intervention in the three groups. According to Tukey’s test, the mean anxiety immediately after the intervention (7–8 cm dilatation) was not significantly different between the two intervention groups (*P* = 0.439), but the mean anxiety was significantly lower than the control group (*P* < 0.001) (Table 3).

**Table 3:** Comparison of mean scores of mothers’ anxiety in three groups of virtual reality, chewing gum and control

Time	Variable	virtual reality	Control	chewing gum	***P*** value
		Mean ± SD	Mean ± SD	Mean ± SD	
**Dilatation 4-5 cm**	**anxiety before intervention**	**40.41 ± 6.32**	**42.45 ± 4.97**	**39.77 ± 6.38**	***P*** = 0.185* **F = 1.720**
	**anxiety immediately after the intervention**	**38.32 ± 6.44**	**44.03 ± 4.90**	**37.48 ± 6.65**	***P*** = 0.000** **Df = 2**
	**anxiety 30 min after intervention**	**38.90 ± 6.12**	**48.48 ± 5.34**	**38.09 ± 5.84**	***P*** = 0.000* **F = 30.957**
**Dilatation 7-8 cm**	**anxiety before intervention**	**43.70 ± 5.67**	**52.19 ± 5.38**	**42.16 ± 4.84**	***P*** = 0.000* **F = 32.048**
	**anxiety immediately after the intervention**	**40.77 ± 6.74**	**54.25 ± 5.88**	**38.90 ± 5.25**	***P*** = 0.000* **F = 60.545**
	**anxiety 30 min after intervention**	**46.12 ± 6.49**	**56.74 ± 5.33**	**43.87 ± 5.10**	***P*** = 0.000** **Df = 2**

*analyzed by ANOVA

**analyzed by Kruskal-Wallis statistical test

In addition, there was a statistically significant difference between anxieties 30 min after intervention in 7-8 cm dilatation in the three study groups. According to Tukey’s test, the mean anxiety 30 min after the intervention (7-8 cm dilatation) was not significantly different between the two intervention groups (*P* = 0.266), but the mean anxiety was significantly lower than the control group (*P* < 0.001) (Table 3). No side effects were reported following chewing gum and watching virtual reality movies Also, after delivery, mothers in intervention groups expressed satisfaction with distraction techniques (chewing gum and virtual reality). As other findings, virtual reality and chewing gum have affected the length of the active phase and the second stage of labor [21].

## Discussion

In the present study, the severity of pain and anxiety of the first stage of childbirth was reduced following the use of the distraction technique (virtual reality and chewing gum).

Pratiw et al.’s study on the “effect of virtual reality on the pain of absurd women” showed that there was a significant difference between pain scores in the VR user group and the non-VR control group (*p* < 0.05) [22]. Also, the results of a study by Wong (2019) on virtual reality in childbirth pain management showed that 57% of the mothers in the study believed that using virtual reality reduced their pain [23, 24].

Several studies have been conducted on the effect of virtual reality on pain outside of childbirth part, all of which point to the positive effect of virtual reality on pain. Study pointed to the effect of virtual reality on threshold and pain tolerance, and the results showed that using virtual reality increased pain threshold and tolerated pain self-efficacy and reduced pain [25].

Two studies on the impact of virtual reality on acute pain [26] and chronic pain [27] both yielded results from the effects of virtual reality on pain. According to studies, the use of virtual reality to reduce pain is widespread in different groups.

The results of our study are in line with the results of the mentioned studies regarding the effect of virtual reality on pain. Limited studies have been reported on the effect of virtual reality on anxiety. The by-results of the virtual reality study in labor pain management showed that 100% of the surveys felt that the use of virtual reality reduces their anxiety [22]. The results of this study is in line with the results of mentioned studies in order to reduce anxiety scores after virtual reality intervention in childbirth stages.

There has been no study to date on the effect of chewing gum on pain intensity in childbirth stages, but various studies have indicated the effect of chewing gum on changes in consciousness, cognitive function, concentration, stress and anxiety.

study stated that chewing gum is a technique for relaxing that improves memory and reduces work stress [28]. A review study by Hirano (2015) on chewing gum and its effect on attention indicated that chewing gum increased attention, increased consciousness, and improved cognitive performance [29]. The results of Ireland study (2016) of comparative evaluation of chewing gum and ibuprofen in orthodontic pain treatment as well as the study (2012) suggest that sugar-free chewing gum may help orthodontic patients to consume less ibuprofen, and chewing gum is effective in reducing pain in orthodontic patients and can be recommended as a suitable alternative to ibuprofen [30, 31]. The results of this study is in line with the results of studies on the effects of chewing gum on pain intensity.

In relation to anxiety, conducted a study aimed at “The effect of chewing gum without sugar on anxiety of active phase of childbirth in nulliparous women”. The results of two study groups with the same anxiety scores were included in the study. According to independent t-test, the anxiety score in the case group was significantly lower than the control group at half an hour after the study [32].. Comparing the results of our study with that of Makvandi’s study, it is evident that subjects with homogeneous anxiety scores were included in the study and changes in anxiety scores were observed after intervention. The results of reducing anxiety 30 min after the intervention are consistent with our study.

In a study (2012) titled “Comparing the effect of chewing mint gum and flavorless gum on stress and anxiety in the first stage of childbirth in nulliparous women”, chewing mint gum and flavorless gum were not significantly different in reducing stress and latent anxiety, but there were significant differences in reducing apparent anxiety level. As a result, chewing mint gum is only more effective in reducing apparent anxiety than flavorless gum [33].

These results are consistent with the results of our study, but are not consistent with the study of (2012) who did not decrease stress after chewing mint gum for 20 min alternatively. According to Gray, the intervention group had a higher level of stress than the control group at the beginning of the study [34], which is inconsistent with our study regarding in terms of homogeneity of anxiety prior to the intervention. Therefore, this discrepancy may be related to the reason for the alternation in chewing mint gum with a given interval.

## Conclusion

This study showed that chewing mint gum alone can reduce the pain of first stage of childbirth in addition to anxiety. In addition, using virtual reality without causing complications can reduce pain and anxiety during childbirth. Therefore, we can use the method of distraction (chewing gum and virtual reality) as painless and anti-anxiety methods that are easy to use and effective in childbirth. This study has several strengths. First, There has been no study to date on the effect of chewing gum on pain intensity in Labor. Second, this is a three-group study designing a clinical trial comparing the use of VR in reducing labor pain and anxiety with chewing gum. The first limitation of the present study was that it was not possible to blind any of the mothers or evaluate them due to their nature. Secondly, non-confirmation of the mothers statements for ethical reasons and thirdly, the effect of individual and genetic characteristics on pain and anxiety tolerance.

## Data Availability

The datasets used and/or analyzed during the current study are available from the corresponding author on reasonable request.

## Abbreviations

VR: Virtual Reality
RCT: Randomized Controlled Trial
VAS: Visual Analogue Scale
IRCT: Iranian Registry of Controlled Trial
